# Broadscale Distribution and Pronounced Mitochondrial Phylogeographic Structure in the Freshwater Minnow *Opsariichthys bidens* Across China's Major River Basins

**DOI:** 10.1002/ece3.74618

**Published:** 2026-11-02

**Authors:** Xinyue Wang, Denghua Yin, Jie Liu, Ming Yan, Wenwen Li, Silei Liu, Min Jiang, Ning Sun, Kai Liu, Yunzhi Yan

**Affiliations:** ^1^ College of Ecology and Environment Anhui Normal University Wuhu China; ^2^ Key Laboratory of Freshwater Fisheries and Germplasm Resources Utilization, Ministry of Agriculture and Rural Affairs, Freshwater Fisheries Research Center Chinese Academy of Fishery Sciences Wuxi China; ^3^ Wuxi Fisheries College Nanjing Agricultural University Wuxi China; ^4^ College of Fisheries and Life Sciences Shanghai Ocean University Shanghai China

**Keywords:** cytochrome b, genetic diversity, mitogenomic features, *Opsariichthys bidens*, phylogeographic structure

## Abstract

China's complex river basins constitute a critical biogeographic setting for investigating genetic differentiation and lineage divergence in freshwater fishes. *Opsariichthys bidens* is broadly distributed among multiple, hydrologically isolated river basins, rendering it a well‐suited system for assessing mitochondrial phylogeographic structure across major river basins. Here, we used newly generated mitochondrial data to characterize broad‐scale genetic variation in *O. bidens* across China's major river basins. We analyzed 379 cytochrome b (*cyt b*) sequences sampled from 13 geographically distinct populations, including seven populations from the Yangtze River and one population from each of six other major rivers, complemented by complete mitochondrial genomes from one individual from each population. Mitochondrial diversity was high at the species level (haplotype diversity, *H*_d_ = 0.942; nucleotide diversity, *π* = 0.02526), but varied markedly among populations, with some populations showing haplotype fixation and others retaining divergent haplotypes. Genetic differentiation was pronounced, with 83.84% of variation attributable to differences among populations; 93.6% of pairwise *F*_st_ values exceeded 0.25. Haplotype‐network and phylogenetic analyses showed two deeply divergent mitochondrial lineages, broadly distributed in northern and southern China, respectively, with further substructure broadly associated with major river basins. Complete mitochondrial genomes exhibited high structural conservation across lineages, and the clearest lineage‐associated annotation differences were observed in the termination codons of *nd1* and *cox3*. Overall, these results provide a broad‐scale mitochondrial phylogeographic assessment of *O. bidens* and reveal a general association between river‐basin geography and mitochondrial lineage structure across China.

## Graphical abstract

This study investigated mitochondrial genetic diversity and phylogeographic structure in *Opsariichthys bidens* across China's major river basins using *cyt b* sequences from 379 individuals and complete mitochondrial genomes from 13 geographic populations. The results revealed limited haplotype sharing, pronounced among‐population differentiation, and two deeply divergent mitochondrial lineages broadly corresponding to northern and southern China. Complete mitogenomes were largely conserved, with the clearest lineage‐associated features observed in the termination codons of *nd1* and *cox3*.

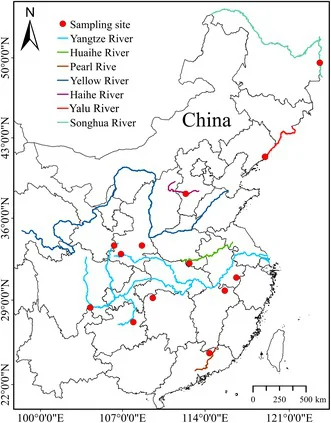

## 1 Introduction

Geographic isolation constitutes a primary driver of biodiversity patterning and remains a cornerstone of phylogeographic research (Avise 2009). Phylogeography integrates the spatial distribution of intraspecific genetic lineages with historical geological and climatic processes, thereby providing a mechanistic framework for elucidating the origins and maintenance of intraspecific differentiation. In freshwater fishes, dispersal and gene flow are often constrained by riverine connectivity and drainage‐basin boundaries, which can promote spatial genetic structure among populations inhabiting different river basins (Bernatchez and Wilson 1998; Ward et al. 1994). East Asia, characterized by its tectonically dynamic river networks and exceptional freshwater fish diversity, offers a model biogeographic setting for investigating drainage‐mediated population differentiation and phylogeographic structuring (Chen et al. 2023). For freshwater fishes occupying multiple major river basins, historical connections among rivers may have facilitated range expansion, whereas contemporary drainage isolation may maintain genetic differentiation among populations. Consequently, such species, exhibiting clear geographic partitioning of mitochondrial lineages, serve as useful natural models for testing hypotheses concerning the concordance between drainage architecture and evolutionary divergence.

*Opsariichthys bidens* is broadly distributed in East Asia, occurring in major Chinese river basins as well as the Korean Peninsula and northern Vietnam (Li et al. 2009; Perdices et al. 2005; Betancur‐R et al. 2017). Mitochondrial investigations have consistently revealed pronounced phylogeographic structure. Perdices et al. (2005) identified deeply divergent mtDNA lineages across the Yangtze, Pearl, and Haihe River basins. Li et al. (2009) resolved two primary mitochondrial clades broadly concordant with northern and southern China, attributing this split to late Cenozoic tectonic uplift and Quaternary climatic oscillations. Fine‐scale studies further corroborate pronounced population‐level genetic structuring within basins (Liu et al. 2023). Collectively, these studies establish *O. bidens* as a suitable system for drainage‐associated evolutionary divergence. Although population‐level mitochondrial variation has been examined in some local studies, a broader evaluation integrating mitochondrial diversity among geographically sampled populations with established lineage‐level phylogeographic patterns remains limited. In particular, uneven geographic coverage and limited sample sizes in some previous studies may constrain reliable interpretation of population‐level mitochondrial diversity among geographic populations. Moreover, although deep divergence has been documented using partial mitochondrial genes, such as *cyt b*, it remains unresolved whether the major lineages exhibit coordinated, mitogenome‐wide differentiation.

To address these gaps, we employed a two‐level mitochondrial approach. We assembled a broad‐scale *cyt b* dataset spanning China's major river basins, with multiple populations sampled within the Yangtze River basin, allowing us to characterize mitochondrial diversity among geographically sampled populations and examine finer scale differentiation within the Yangtze River basin in the context of broader mitochondrial lineage patterns. Complementing this, we generated complete mitochondrial genomes from one individual from each sampled population. Whole mitogenomes offer richer character sets than individual gene fragments and have been widely used for comparative and phylogenetic analyses (Wang et al. 2022; Liu et al. 2025; Camacho et al. 2022; Papetti et al. 2021; Duchêne et al. 2011). However, mitogenomic comparisons among geographic populations or divergent lineages of *O. bidens* remain scarce, despite the availability of a reference mitogenome (Wang et al. 2007). By integrating these two data levels, our design enables an examination of whether the deep mitochondrial divergence known from fragment‐based studies extends to lineage‐associated mitogenomic features, bridging the gap from population haplotype variation to whole‐mitogenome architecture.

In this study, we sampled 13 geographic populations of *O. bidens* from China's major river basins, obtained *cyt b* sequences from 379 individuals, and analyzed complete mitochondrial genomes from one individual from each sampled population. Specifically, we aimed to: (1) characterize mitochondrial diversity among geographic populations across China's major river basins and assess finer‐scale differentiation within the Yangtze River basin in relation to the broader phylogeographic structure; and (2) evaluate whether deep mitochondrial lineage divergence is reflected in detectable mitogenomic features. By addressing these objectives, this study provides new mitochondrial evidence for understanding phylogeographic divergence in *O. bidens* and offers a basis for future studies integrating nuclear genomic and biogeographic data.

## 2 Materials and Methods

### 2.1 Ethics Statement

All animal handling and sampling procedures complied with the Laboratory Animal Welfare and Ethics Review Guide (GB/T 35892‐2018) issued by the Technical Committee on Laboratory Animal Science of the Standardization Administration of China (SAC/TC 281). All animal experiments were approved by the Experimental Management and Ethics Committee of Anhui Normal University (Approval No. AHNU‐ET2022023).

### 2.2 Sample Collection and Preservation

From 2023 to 2025, a total of 379 *O. bidens* specimens were collected from 13 geographic populations spanning seven major rivers in China (Table 1, Figure 1), including the Yangtze River (YZ1‐YZ7), Pearl River (PR), Yellow River (YR), Huaihe River (HUR), Haihe River (HAR), Yalu River (LR), and Songhua River (SR). Preliminary identification used lateral stripe patterns and the distinct notch at the anterior tip of the upper lip to distinguish the specimens from *Zacco* and from *Opsariichthys* congeners lacking this notch (Chen et al. 2009; Peng et al. 2024). Morphological identification was supplemented by *cyt b* phylogenetic comparison with GenBank reference sequences (Table S1). Muscle or fin tissue was collected according to field conditions and specimen preservation needs and preserved in ethanol for DNA extraction. The *cyt b* was sequenced for all 379 individuals. Complete mitochondrial genomes were obtained from whole‐genome resequencing data for one individual from each population (*n* = 13), selected based on sequencing quality.

**TABLE 1:** Sampling information for *O. bidens* from different river basins.

River system	City, province	Sample size	Population ID	Latitude (N)	Longitude (E)
Yangtze River	Yibin, Sichuan	32	YZ1	28.74622	104.14521
	Baoji, Shaanxi	10	YZ2	34.05003	106.56135
	Shiqian, Guizhou	31	YZ3	27.45991	108.22388
	Sangzhi, Hunan	30	YZ4	29.41413	110.20482
	Hanzhong, Shaanxi	32	YZ5	33.29978	107.25218
	Jingdezhen, Jiangxi	29	YZ6	29.35251	117.21500
	Xuancheng, Anhui	29	YZ7	30.29010	118.53514
Pearl River	Heyuan, Guangdong	31	PR	24.34382	114.96923
Yellow River	Xi'an, Shaanxi	29	YR	33.95799	109.44546
Songhua River	Shuangyashan, Heilongjiang	32	SR	46.51472	133.85898
Haihe River	Shijiazhuang, Hebei	32	HAR	38.10770	114.60760
Yalu River	Dandong, Liaoning	30	LR	40.04344	124.13528
Huaihe River	Xinyang, Henan	32	HUR	32.06812	114.10947

**FIGURE 1:**
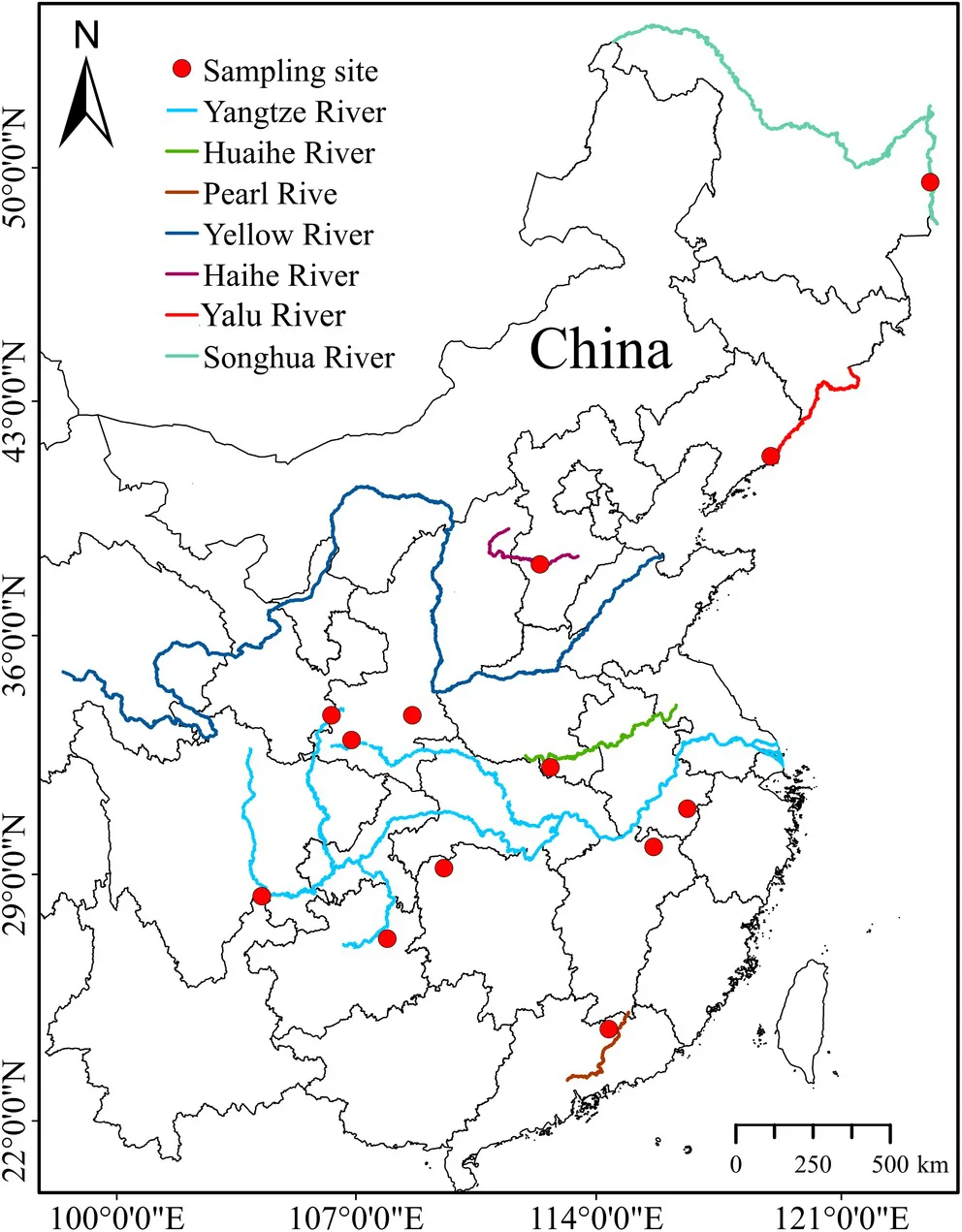
Sampling sites of *O. bidens* .

### 2.3 DNA Extraction and *cyt b* Amplification

Genomic DNA was extracted from muscle or fin clips using the Ezup Column Animal Genomic DNA Kit (Sangon Biotech, China) following the manufacturer's protocol. DNA integrity was evaluated by agarose gel electrophoresis, and DNA concentration and purity were measured using a spectrophotometer. The *cyt b* gene was amplified by PCR with primers JtsF (5′‐AACCACCGTTGTAATTCAACTA‐3′) and JtsR (5′‐TAGTTTAGTTTAGAATTCTGGCTTTGG‐3′) (Hardman and Page 2003). Each 25 μL PCR reaction contained 2.5 μL 10× Ex Taq Buffer (Mg^2+^ plus, 20 mmol/L), 2 μL dNTP mix (2.5 mmol/L), 0.25 μL Ex Taq DNA polymerase (5 U/μL, TaKaRa, Japan), 0.5 μL of each primer (10 μmol/L), 1 μL template DNA, and double‐distilled water to volume. The PCR program consisted of an initial denaturation at 95°C for 5 min; 33 cycles of denaturation at 94°C for 30 s, annealing at 52.7°C for 30 s, and extension at 72°C for 45 s; followed by a final extension at 72°C for 10 min. PCR products were submitted to a commercial service provider for bidirectional Sanger sequencing.

### 2.4 Mitochondrial Genome Assembly and Annotation

Genomic DNA libraries with an average insert size of approximately 350 bp were prepared and sequenced using cPAS technology. Following read quality control, mitochondrial genomes were assembled with NOVOPlasty (v 4.3.5) (Dierckxsens et al. 2016). Genome annotation was performed using the MITOS web server (Bernt et al. 2013). The resulting annotations were further inspected by comparison with closely related species, and manual refinements were applied when necessary to obtain the final annotation set.

### 2.5 Genetic Differentiation Analysis Based on *cyt b*

The *cyt b* sequences were inspected and edited in BioEdit (v 7.0.9; http://www.mbio.ncsu.edu/bioedit/bioedit.html), and alignments were generated using ClustalX (v 1.83) (Thompson 1997) to obtain a consensus sequence length. MEGA (v 7.0) (Kumar et al. 2016) was used to calculate nucleotide composition and Kimura 2‐parameter genetic distances among populations and to construct a neighbor‐joining (NJ) tree. Node support was assessed with 1000 bootstrap replicates. An additional maximum likelihood (ML) analysis was performed to assess the robustness of the NJ groupings. Reference *cyt b* sequences from closely related *Opsariichthys* species, *Zacco platypus* and *Candidia barbatus* were included in the phylogenetic analysis used for molecular‐assisted identification (Figure 4; Table S1).

A haplotype network was reconstructed in PopART (Leigh and Bryant 2015). Genetic diversity indices, including the number of variable sites, number of haplotypes (*h*), haplotype diversity (*H*_d_), nucleotide diversity (*π*), and the average number of nucleotide differences (*k*), were estimated in DnaSP (v 6.0) (Rozas et al. 2017). Arlequin (v 3.5) (Excoffier and Lischer 2010) was used to perform AMOVA, estimate the fixation index (*F*_st_), and conduct neutrality tests (Tajima's *D* and Fu's *F*_s_). Pairwise *F*_st_ significance was assessed by 9999 permutations between populations with Benjamini–Hochberg correction. 95% percentile CIs were estimated from 10,000 bootstrap resamples of individuals within populations, preserving sample sizes. For the seven Yangtze River populations, sampling coordinates were snapped to the nearest HydroRIVERS reach, and pairwise distances were calculated as the shortest paths along the river network (Lehner and Grill 2013). Isolation by distance was assessed using a one‐sided exact Mantel test based on Pearson correlation, enumerating all 5040 population‐label permutations to test for a positive association. Raw *F*_st_ was used because one pair had *F*_st_ = 1.000.

### 2.6 Mitogenome‐Based Phylogenetic and Comparative Sequence Analyses

The nucleotide sequences of the 13 protein‐coding genes (PCGs) were extracted and concatenated in PhyloSuite (v 2.0) (Zhao et al. 2025). The phylogeny based on the concatenated 13 PCGs was reconstructed in MEGA (v 7.0) using the same NJ/K2P procedure with 1000 bootstrap replicates, using *Zacco platypus* as the outgroup. Nucleotide composition, amino acid usage frequencies, and relative synonymous codon usage (RSCU) were calculated in MEGA (v 7.0) (Kumar et al. 2016). Strand asymmetry in nucleotide composition was quantified using the skew formula. For each of the 13 PCGs, DnaSP (v 6.0) (Rozas et al. 2017) was used to estimate the numbers of conserved and variable sites, the rates of non‐synonymous (dN) and synonymous (dS) substitutions, and their ratio Ka/Ks (dN/dS). Mean pairwise nucleotide differences were calculated as uncorrected p‐distances separately for each PCG and for the concatenated alignments including and excluding *cyt b* across the same 13 mitogenome individuals. Columns containing gaps or ambiguous bases in any individual were excluded, and the values were averaged across all 78 pairwise comparisons without including the outgroup.

## 3 Results

### 3.1 Genetic Diversity and Neutrality Tests of *O. bidens*

Genetic diversity was evaluated for 13 geographic populations of *O. bidens* distributed across major rivers in China. In total, 379 *cyt b* sequences were obtained and aligned, producing a 1025 bp dataset. The alignment contained 138 variable sites, including 120 parsimony‐informative sites and 18 singleton variable sites. Overall nucleotide composition was biased, with A + T content (53.7%) exceeding G + C content (46.3%). We identified 66 haplotypes across all individuals. At the species level, haplotype diversity (*H*_d_), nucleotide diversity (*π*), and the average number of nucleotide differences (*k*) were 0.942, 0.02526, and 25.839, respectively, indicating high haplotype diversity (*H*_d_ > 0.8) and high nucleotide diversity (*π* > 0.01).

Genetic diversity varied markedly among populations (Table 2). *H*_d_ ranged from 0 to 0.889 and *π* ranged from 0 to 0.02363. The YR population showed the highest nucleotide diversity (*π* = 0.02363, *k* = 24.202), whereas both *H*_d_ and *π* were zero in YZ1 and YZ3, indicating complete haplotype identity within these populations. Overall, mitochondrial diversity was high at the species level but unevenly distributed among populations.

**TABLE 2:** Population genetic diversity parameters based on *cyt b* gene sequences.

Groups	Number of variable sites (*S*)	Number of haplotypes (*h*)	Haplotype diversity (*H*_d_)	Nucleotide diversity (*π*)	Average number of nucleotide differences (*k*)
YZ1	0	1	0.000	0.00000	0.000
YZ2	7	4	0.644	0.00293	3.000
YZ3	0	1	0.000	0.00000	0.000
YZ4	4	3	0.191	0.00032	0.329
YZ5	8	6	0.808	0.00243	2.490
YZ6	49	9	0.837	0.00692	7.084
YZ7	56	10	0.781	0.01485	15.202
PR	5	6	0.400	0.00043	0.439
YR	50	3	0.626	0.02363	24.202
SR	10	10	0.609	0.00078	0.796
HAR	43	11	0.889	0.00378	3.879
LR	8	3	0.191	0.00058	0.595
HUR	9	7	0.681	0.00136	1.395

Populations exhibited different combinations of mitochondrial diversity, including low *H*_d_ with low *π*, high *H*_d_ with low *π*, and high *H*_d_ with high *π*. Tajima's *D* ranged from −2.32939 to 3.37761 and Fu's *F*_s_ ranged from −8.15454 to 29.27516 across populations (Table 3).

**TABLE 3:** Neutrality test of *O. bidens* populations based on *cyt b*.

Groups	Tajima's *D*	*p*	Fu's *F*_s_	*p*
YZ1	0.00000	1.000	0.00000	N.A.
YZ2	0.89971	0.834	1.54834	0.784
YZ3	0.00000	1.000	0.00000	N.A.
YZ4	−1.73164	0.024	−0.73084	0.200
YZ5	0.75859	0.800	1.20824	0.757
YZ6	−1.61904	0.050	2.55888	0.853
YZ7	0.24903	0.667	6.22861	0.974
PR	−1.75710	0.019	−4.36765	0.000
YR	3.37761	1.000	29.27516	1.000
SR	−2.12120	0.001	−8.15454	0.000
HAR	−2.32939	0.001	−1.04280	0.385
LR	−2.14213	0.000	0.41605	0.546
HUR	−1.15082	0.126	−1.49190	0.170

### 3.2 Genetic Differentiation and Population Structure of *O. bidens*

Population genetic structure was assessed for the same 13 populations across major rivers in China. AMOVA showed that differences among populations accounted for 83.84% of total genetic variation, with an overall *F*_st_ of 0.838 (Table 4). This indicates that genetic variation is predominantly partitioned among populations and confirms a strong population genetic structure.

**TABLE 4:** Results of AMOVA for *O. bidens* populations based on *cyt b*.

Source of variation	df	Sum of square	Variance component	Percentage of variation
Among populations	12	4066.611	11.58375	83.84
Within populations	366	816.996	2.23223	16.16
Total	378	4883.607	13.81598	
*F*_st_ = 0.838				

Pairwise comparisons further supported extensive differentiation. Mean between‐population K2P genetic distances were ≥ 0.020 in 76.9% of population pairs, indicating substantial divergence (Table S2). Consistently, 93.6% of pairwise *F*_st_ values were greater than 0.25 (Figure 2), meeting the criterion for high genetic differentiation (Wright 1990). All population pairs except YZ3‐YZ4 (adjusted *p* = 0.112) showed significant differentiation after correction (adjusted *p* < 0.05; Table S3). Haplotype distributions were highly population‐specific: private haplotypes comprised 89.4% of all haplotypes detected (Figure 3, Table S4). Collectively, these patterns demonstrate pronounced and widespread genetic differentiation in *O. bidens* at the national scale. Within the Yangtze River basin, *F*_st_ was not significantly positively correlated with river distance (Mantel *r* = −0.300, *p* = 0.912; Figure S1).

**FIGURE 2:**
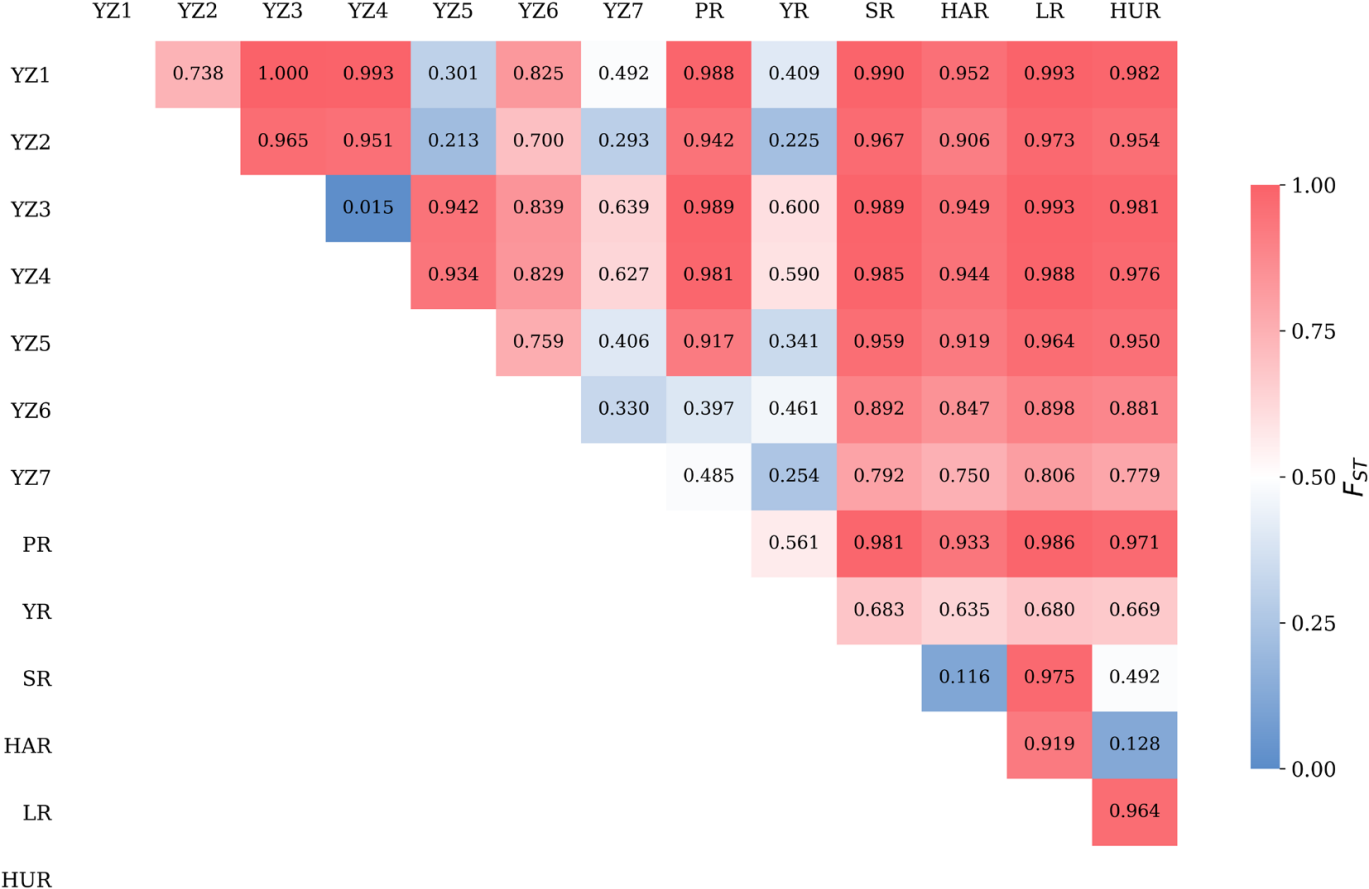
Heatmap of *F*_st_ among different geographic populations of *O. bidens* . Values are rounded to three decimal places; uncertainty estimates and significance tests are provided in Table S3.

**FIGURE 3:**
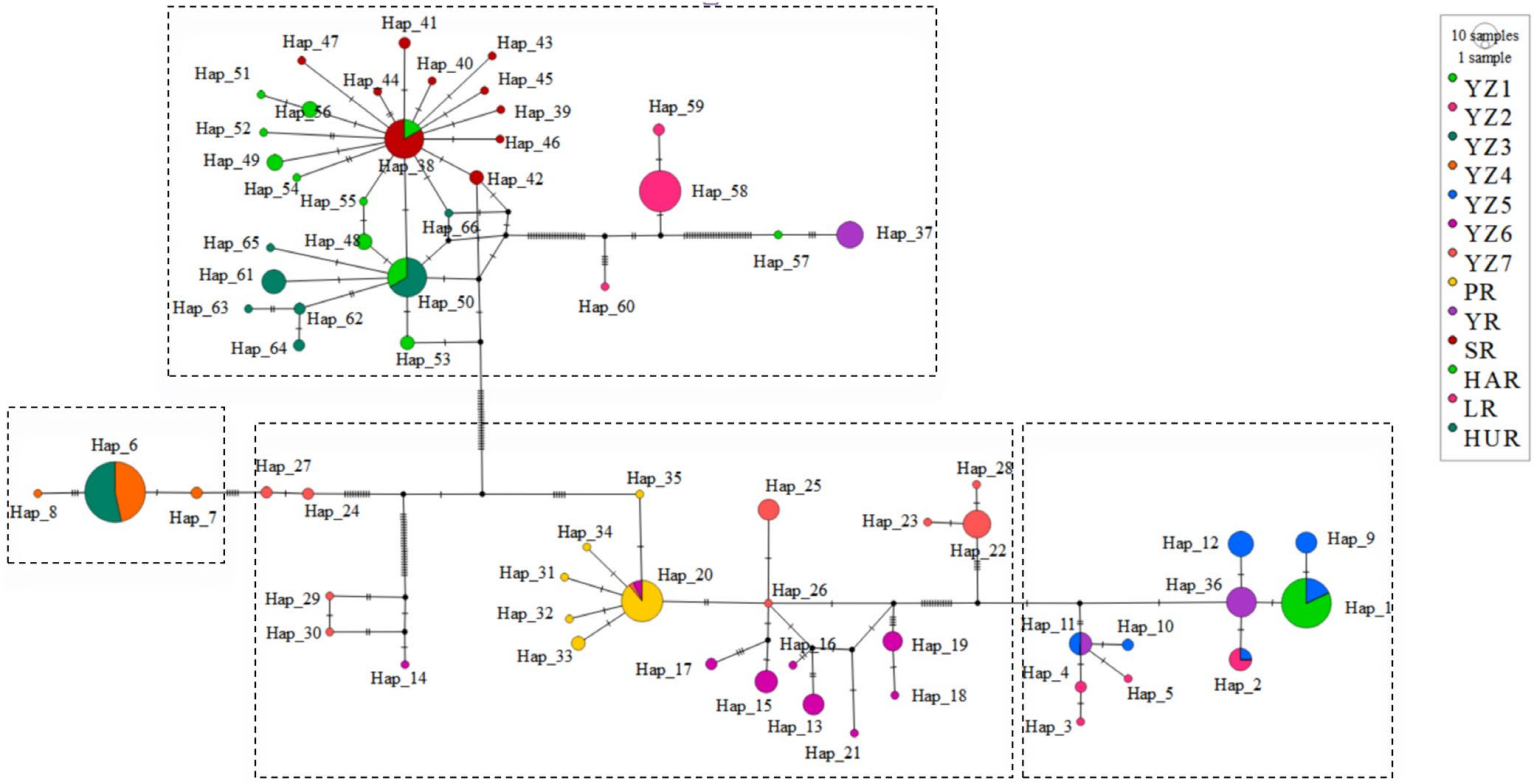
Haplotype network of *O. bidens* based on *cyt b* gene sequences.

Both the haplotype network and the phylogenetic tree (Figure 4) supported two main lineages with a broad north–south geographic pattern. Most Yangtze River individuals and the Pearl River samples were placed in the southern lineage. Three Yangtze River individuals (YZ6‐54, YZ7‐37, and YZ7‐38) grouped with the northern samples, but this association received only 46.3% bootstrap support. ML recovered the main sample groups but not the complete NJ southern clade; the three individuals' northern placement remained weakly supported (43.3%; Figure S2). Their lineage placement was therefore treated as unresolved. Within the southern lineage, additional substructure was evident and broadly matched geography. Specifically, an upper Yangtze group was primarily represented by YZ1 and YZ2, a middle Yangtze group by YZ3 and YZ4, and a lower Yangtze plus Pearl River group by YZ6, YZ7, and PR. Notably, the YR population did not form a single exclusive branch in the phylogenetic tree. YR also shared haplotype Hap11 with YZ5, suggesting a possible contact signal between northern and southern mitochondrial lineages.

**FIGURE 4:**
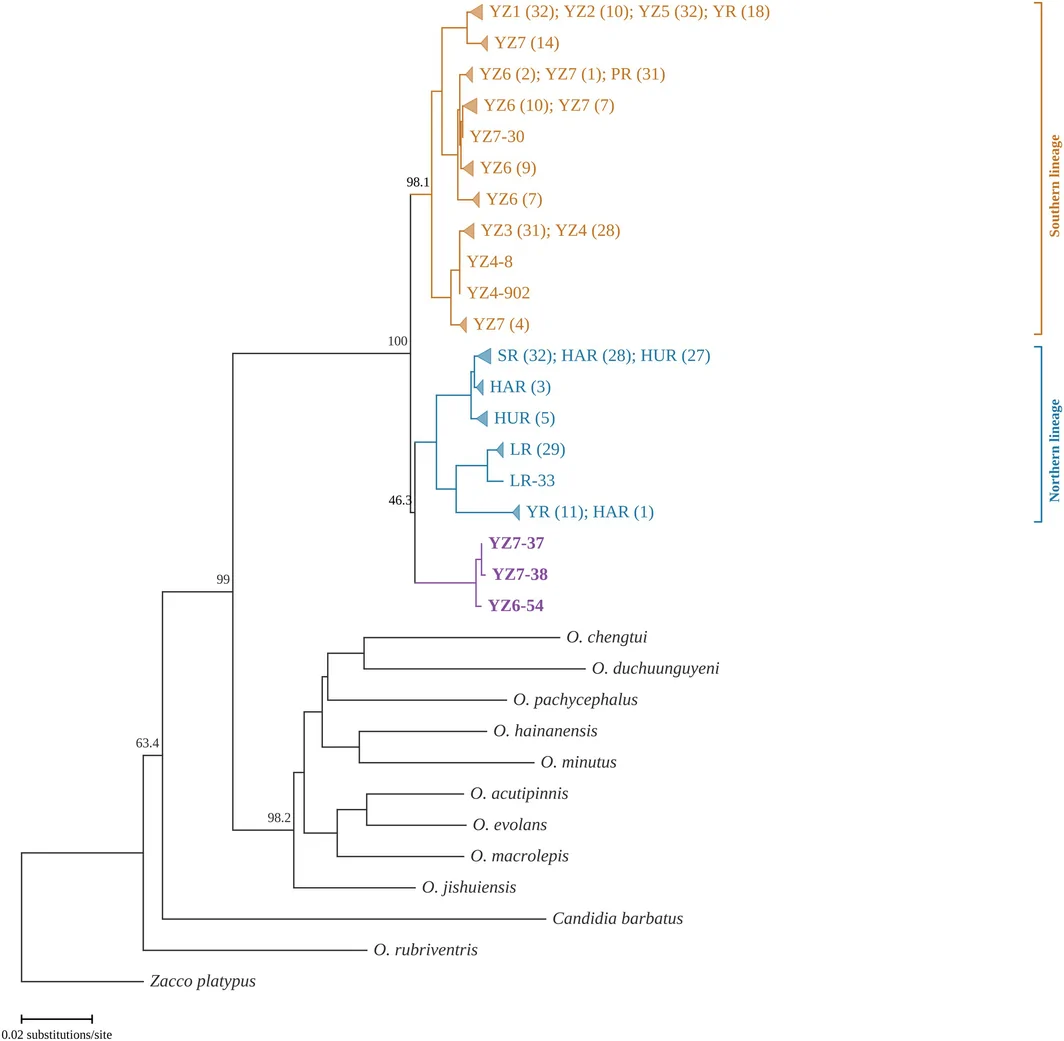
Phylogenetic tree of *O. bidens* and reference taxa constructed based on *cyt b* gene sequences. Triangles indicate collapsed clades; numbers in parentheses give the number of study individuals represented. Bootstrap support values are shown at selected nodes.

### 3.3 Characteristics of the Mitochondrial Genome

We further compared complete mitochondrial genomes representing the northern and southern lineages. Across the 13 sampled individuals, mitochondrial genomes were highly conserved in gene content and gene order (Figure 5A). Each genome contained 13 PCGs, 22 transfer RNA genes (tRNAs), two ribosomal RNA genes (rRNAs), and one control region (CR). No gene rearrangements or gene losses were detected. Mitochondrial genome length ranged from 16,610 to 16,613 bp, and the PCG region spanned 11,223 to 11,457 bp. Overall AT content was relatively high, and differences in base composition among populations were small.

**FIGURE 5:**
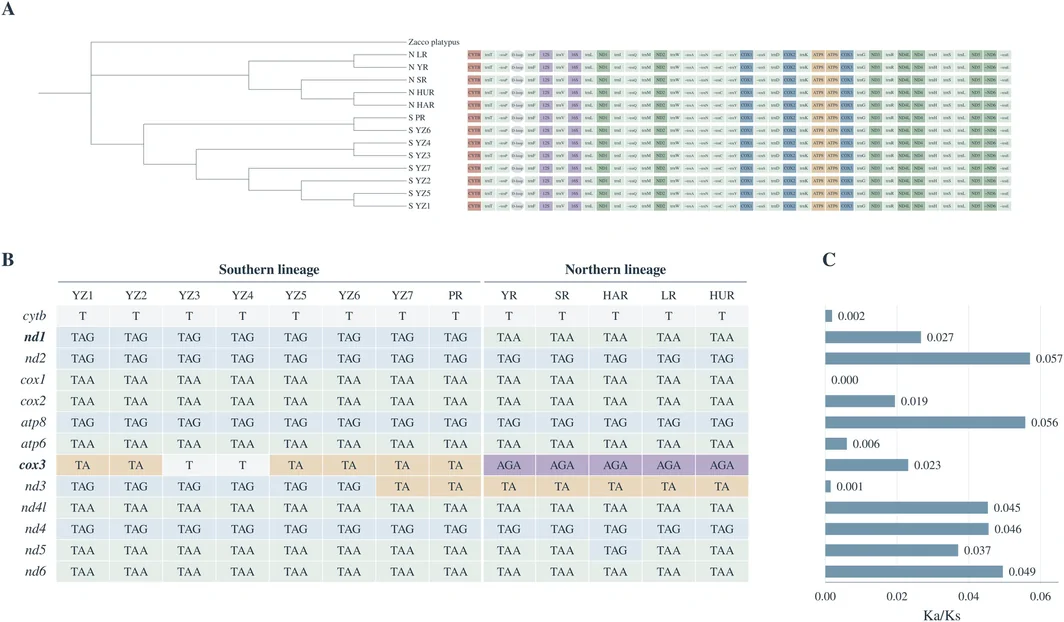
(A) Mitogenome organization and phylogenetic relationships based on the concatenated 13 PCGs; (B) Termination codon usage; (C) Selection pressure analysis of protein‐coding genes.

Mean pairwise nucleotide differences ranged from 1.352% to 5.204% among PCGs, with *cyt b* intermediate at 2.743%; concatenated values including and excluding *cyt b* were 3.007% and 3.034%, respectively (Table S5). All 13 PCGs showed Ka/Ks values below 1 (Figure 5C), consistent with predominant purifying selection. Amino acid composition was also broadly similar among sampled mitogenomes (Figure S3).

Comparison between the sampled northern and southern mitochondrial lineages showed that overall base composition was similar between the northern and southern lineages. The northern lineage showed slightly stronger compositional bias than the southern lineage (Table S6), but this difference was limited. Codon usage patterns in PCGs were generally conserved, and start codons were highly consistent across populations. Among the sampled mitogenomes, the clearest lineage‐associated annotation differences involved *nd1* (TAG in southern vs. TAA in northern samples) and *cox3* (TA/T vs. AGA) (Figure 5B). RSCU analyses showed minor differences in synonymous codon preferences between the northern and southern lineages (Figure S4), but these differences were less pronounced than the termination codon differences.

## 4 Discussion

### 4.1 Genetic Diversity and Genetic Differentiation of *O. bidens*

Using *cyt b* sequences from *O. bidens* sampled across major Chinese rivers, we detected pervasive and strong mitochondrial differentiation among populations. This pattern is consistent with the species' habitat preferences and ecological characteristics (Zhong et al. 2020). As a resident freshwater fish inhabiting streams and tributaries, *O. bidens* has limited capacity for movement across drainage divides, and dispersal is largely constrained by river network connectivity (Fagan 2002). When gene flow among populations is reduced or interrupted for extended periods, genetic differences are expected to accumulate through time (Avise 2000; Bernatchez and Wilson 1998). The pronounced differentiation observed here conforms to this general expectation. Previous *cyt b* studies attributed 93% and 96% of variation to differences among populations (He et al. 2025; Li et al. 2009), compared with 83.84% here, indicating strong population subdivision across studies with different geographic and taxonomic coverage. Beyond confirming the broad mitochondrial differentiation patterns reported in previous studies, our findings further reveal substantial variation in genetic differentiation among geographic populations, particularly among the seven populations within the Yangtze River basin.

Differentiation varied among population pairs: within the Yangtze River basin, *F*_st_ ranged from 0.015 (YZ3–YZ4) to 1.000 (YZ1–YZ3), with no significant positive isolation‐by‐distance association detected among these seven populations. At the broader geographic scale, the major mitochondrial lineages and their substructure were broadly aligned with watershed boundaries and regional geographic divisions, which is consistent with the expectation that riverine landscapes and long‐term drainage isolation can shape phylogeographic patterns in freshwater fishes (Avise 2000; Bernatchez and Wilson 1998). The broad north–south mitochondrial pattern roughly corresponds to the Qinling Mountains–Huaihe River line, with geographic overlap between lineages (Avise 2000; Hewitt 2000). The separation of northern populations from most Yangtze and Pearl River samples broadly resembles that reported by Lin et al. (2016), although lineage boundaries differ. At smaller spatial scales, lineage subdivisions also showed spatial agreement with regional watershed units. For example, the Nanling Mountains separate the Yangtze and Pearl river basins and have been discussed as an important barrier in freshwater fish phylogeographic studies (Yang et al. 2012). In northern China, low relief divides and mountainous terrain may also contribute to drainage isolation and lineage divergence in freshwater fishes (Ni et al. 2023). These geographic patterns suggest that drainage configuration is associated with the spatial distribution of mitochondrial lineages in *O. bidens* , although direct barrier effects require further testing with landscape genetic or biogeographic evidence.

Genetic diversity also varied markedly among geographic populations, ranging from haplotype fixation to high within‐population diversity. Such heterogeneity may reflect differences in local demographic history, population size, habitat connectivity, and long‐term isolation among basins and local populations (Grant 1998; Nei et al. 1975). The low diversity observed in YZ3 (Shiqian, Guizhou) may be associated with local isolation in a fragmented upland drainage landscape. A similar reduction in diversity was observed in YZ1 (Yibin, Sichuan), which may also reflect restricted connectivity and enhanced effects of genetic drift in local drainage habitats. In such populations, reduced effective population size and limited gene flow can promote haplotype fixation and the loss of genetic variation (Whitlock 2000; Frankham 2003). In contrast, the high diversity and haplotype sharing observed in YR may indicate a more complex mitochondrial history near the north–south transition zone. River network structure and habitat heterogeneity can influence connectivity and dispersal routes in fishes (Davis et al. 2018), but mitochondrial data alone cannot distinguish among historical admixture, incomplete lineage sorting, and recent gene flow. Therefore, these local patterns should be further examined using nuclear genomic and biogeographic evidence.

Overall, these results suggest that historical drainage connectivity and long‐term basin isolation may have jointly shaped the present mitochondrial differentiation pattern of *O. bidens* . Under the combined influence of China's stepped topography and fragmented drainage systems, *O. bidens* shows a strongly structured pattern of mitochondrial differentiation and marked heterogeneity in genetic diversity among populations.

### 4.2 Mitochondrial Genome Characteristics and Lineage Differentiation Signals of *O. bidens*

The apparent discrepancy concerns different aspects of mitochondrial variation: pronounced *cyt b* population structure does not imply unusually high nucleotide divergence, as *cyt b* variation (2.743%) was below that of the remaining 12 PCGs (3.034%). Geographic isolation and drift may differentiate maternal haplotype frequencies, while purifying selection constrains nonsynonymous changes, with Ka/Ks values typically below 1 (Zhao et al. 2022). These processes can produce strong geographic structure alongside coding conservation, although their relative contributions remain unresolved; one mitogenome per population also limits inference about within‐population variation.

Despite overall conservation, the annotations of the sampled mitogenomes showed consistent differences in termination codon usage for *nd1* and *cox3* between the northern and southern lineages, whereas start codons remained highly consistent across populations. One plausible explanation is that incomplete mitochondrial stop codons can be completed through posttranscriptional processes, potentially reducing functional constraints on stop codon variants. Under long‐term geographic isolation, such variants may accumulate and contribute to lineage‐associated mitogenomic differences (Nie et al. 2021). Consistently, Li et al. (2024) reported that termination codon types can be replaced in specific genes within certain lineages, indicating that such changes can contribute to lineage‐level differentiation patterns.

Lineage‐level differences in base composition and synonymous codon usage were also detected, but these differences were weak and mainly involved minor shifts at third codon positions. Because third codon positions are generally less constrained and can accumulate substitutions more readily (Goios et al. 2007), such patterns may provide supplementary information on lineage divergence. However, compared with the termination codon differences in *nd1* and *cox3*, base‐composition and RSCU shifts were less pronounced and should not be overemphasized as major lineage‐diagnostic features. Previous comparative mitogenomic work in *Opsariichthys* and related taxa also supports the usefulness of mitochondrial sequence features for evolutionary comparison (Wang et al. 2007).

In summary, complete mitogenomes of the northern and southern lineages were conserved in gene content and order, while exhibiting lineage‐associated sequence variation. Among these features, the current annotations showed differences in termination codon usage of *nd1* and *cox3*, whereas base composition and synonymous codon usage showed only minor shifts. Together, these results reveal geographically structured maternal variation alongside conserved gene organization and constrained coding evolution, with lineage‐associated termination codon differences.

## 5 Conclusions

This study confirmed high mitochondrial diversity, strong among‐population differentiation, and a broad north–south mitochondrial pattern with geographic overlap, with additional river‐basin‐related substructure. Complete mitogenomes were largely conserved, and the clearest lineage‐associated features were differences in the termination codons of *nd1* and *cox3*. Pronounced maternal population structure thus coexists with conserved mitogenomic organization and coding‐sequence patterns consistent with purifying selection. Further studies incorporating broader mitogenomic sampling and nuclear genomic data would help clarify within‐population variation and the evolutionary processes underlying the observed mitochondrial patterns. This study provides a mitochondrial reference for future genomic and biogeographic studies of phylogeographic divergence in *O. bidens* across China's major river basins.

## Author Contributions

**Xinyue Wang:** conceptualization (equal), data curation (equal), formal analysis (equal), writing – original draft (equal). **Denghua Yin:** validation (equal), writing – review and editing (equal). **Jie Liu:** investigation (equal), resources (equal), writing – review and editing (equal). **Ming Yan:** data curation (equal), formal analysis (equal), validation (equal). **Wenwen Li:** investigation (supporting), resources (supporting), writing – review and editing (supporting). **Silei Liu:** investigation (equal), resources (equal), writing – review and editing (equal). **Min Jiang:** investigation (equal), resources (equal), writing – review and editing (equal). **Ning Sun:** project administration (equal), validation (equal). **Kai Liu:** conceptualization (equal), project administration (equal), writing – review and editing (equal). **Yunzhi Yan:** conceptualization (equal), supervision (equal), writing – review and editing (equal).

## Funding

This work was funded by the Central Public‐Interest Scientific Institution Basal Research Fund, CAFS (No. 2023TD11), Monitoring of Aquatic Resources in Key Waters of Anhui Province” (Project No. 2024BFAFZ02936), and the Youth Project of Anhui Provincial Natural Science Foundation (No. 2408085QC107).

## Conflicts of Interest

The authors declare no conflicts of interest.

## Supporting information


**Figure S1:** Pairwise *F*_st_ versus river‐network distance among seven Yangtze River populations.
**Figure S2:** Maximum likelihood tree of *O. bidens* and reference taxa based on *cyt b* sequences. Triangles indicate collapsed clades; numbers in parentheses give the number of study individuals represented. Bootstrap support values are shown at selected nodes.
**Figure S3:** Heatmap of amino acid composition ratios.
**Figure S4:** Heatmap of synonymous codon usage.
**Table S1:** GenBank reference sequences used in the *cyt b* phylogenetic analysis.
**Table S2:** Pairwise K2P genetic distances within and between *O. bidens* populations based on *cyt b* sequences.
**Table S3:** Pairwise mitochondrial differentiation among *O. bidens* populations based on *cyt b*.
**Table S4:** Haplotype distribution in *O. bidens* populations based on *cyt b*.
**Table S5:** Mean pairwise nucleotide differences and Ka/Ks values of the 13 PCGs in *O. bidens*.
**Table S6:** The base composition and base preference of mitochondrial genomes in different geographical populations of *O. bidens*.

## Data Availability

All newly generated *cytochrome b* gene sequences and complete mitochondrial genomes from this study have been deposited in GenBank under accession numbers PZ103026–PZ103404 and PZ113238–PZ113250.
