# Supplementary material for: Broadscale Distribution and Pronounced Mitochondrial Phylogeographic Structure in the Freshwater Minnow Opsariichthys bidens Across China's Major River Basins
Source: Ecol Evol. 2026 Nov 2;16(11):e74618. doi: 10.1002/ece3.74618 (PMC13727371; doi:10.1002/ece3.74618)
Supplement: Supplementary file 1 [file ECE3-16-e74618-s001.docx]

**Supplementary Materials**

# **Figure Legends**

**Supplementary Figure S1** Pairwise *F_st_* versus river-network distance among seven Yangtze River populations. Significance was assessed using a one-sided exact Mantel test.

**Supplementary Figure S2** Maximum-likelihood tree of *O. bidens* and reference taxa based on *cyt b* sequences.

**Supplementary Figure S3** Heatmap of amino acid composition ratios.

**Supplementary Figure S4** Heatmap of synonymous codon usage.

# Table Legends

**Supplementary Table S1** GenBank reference sequences used in the *cyt b* phylogenetic analysis.

**Supplementary Table S2** Pairwise K2P genetic distances within and between *O. bidens* populations based on *cyt b* sequences.

**Supplementary Table S3** Pairwise mitochondrial differentiation among *O. bidens* populations based on *cyt b*.

**Supplementary Table S4** Haplotype distribution in *O. bidens* populations based on *cyt b.*

**Supplementary Table S5** Mean pairwise nucleotide differences and Ka/Ks values of the 13 PCGs in *O. bidens.*

**Supplementary Table S6** The base composition and base preference of mitochondrial genomes in different geographical populations of *O. bidens.*

Supplementary Figure S1 Pairwise *F_st_* versus river-network distance among seven Yangtze River populations.

Supplementary Figure S2 Maximum-likelihood tree of *O. bidens* and reference taxa based on *cyt b* sequences.

Triangles indicate collapsed clades; numbers in parentheses give the number of study individuals represented. Bootstrap support values are shown at selected nodes

Supplementary Figure S3 Heatmap of amino acid composition ratios.

Supplementary Figure S4 Heatmap of synonymous codon usage.

Supplementary Table S1. GenBank reference sequences used in the *cyt b* phylogenetic analysis.

| Species | GenBank accession | Source record |
| --- | --- | --- |
| *Opsariichthys chengtui* | KT725244.1 | Complete mitogenome |
| *Opsariichthys macrolepis* | PZ630798.1 | Complete mitogenome |
| *Opsariichthys rubriventris* | PX122525.1 | *cyt b* sequence |
| *Opsariichthys duchuunguyeni* | KJ940956.1 | *cyt b* sequence |
| *Opsariichthys pachycephalus* | KR698649.1 | *cyt b* sequence |
| *Opsariichthys acutipinnis* | KJ940954.1 | *cyt b* sequence |
| *Opsariichthys evolans* | MH350437.1 | *cyt b* sequence |
| *Opsariichthys jishuiensis sp. nov* | PV929876.1 | Complete mitogenome |
| *Opsariichthys hainanensis* | KJ940933.1 | *cyt b* sequence |
| *Zacco platypus* | PP529841.1 | *cyt b* sequence |
| *Candidia barbatus* | AY958200.1 | *cyt b* sequence |
| *Opsariichthys minutus* | KR698540.1 | *cyt b* sequence |

For reference records comprising complete mitochondrial genomes, only the annotated *cyt b* sequences were extracted and used for phylogenetic analysis.

Supplementary Table S2 Pairwise K2P genetic distances within and between *O. bidens* populations based on *cyt b* sequences.

| Groups | YZ1 | YZ2 | YZ3 | YZ4 | YZ5 | YZ6 | YZ7 | PR | YR | SR | HAR | LR | HUR |
| --- | --- | --- | --- | --- | --- | --- | --- | --- | --- | --- | --- | --- | --- |
| YZ1 | 0.000 |  |  |  |  |  |  |  |  |  |  |  |  |
| YZ2 | 0.003 | 0.003 |  |  |  |  |  |  |  |  |  |  |  |
| YZ3 | 0.022 | 0.020 | 0.000 |  |  |  |  |  |  |  |  |  |  |
| YZ4 | 0.022 | 0.020 | 0.000 | 0.000 |  |  |  |  |  |  |  |  |  |
| YZ5 | 0.002 | 0.003 | 0.022 | 0.022 | 0.002 |  |  |  |  |  |  |  |  |
| YZ6 | 0.019 | 0.019 | 0.021 | 0.021 | 0.020 | 0.007 |  |  |  |  |  |  |  |
| YZ7 | 0.014 | 0.015 | 0.021 | 0.021 | 0.015 | 0.017 | 0.015 |  |  |  |  |  |  |
| PR | 0.018 | 0.018 | 0.020 | 0.020 | 0.018 | 0.006 | 0.015 | 0.000 |  |  |  |  |  |
| YR | 0.020 | 0.020 | 0.030 | 0.030 | 0.020 | 0.029 | 0.027 | 0.028 | 0.025 |  |  |  |  |
| SR | 0.041 | 0.040 | 0.039 | 0.039 | 0.041 | 0.036 | 0.038 | 0.033 | 0.039 | 0.001 |  |  |  |
| HAR | 0.041 | 0.040 | 0.039 | 0.039 | 0.040 | 0.036 | 0.038 | 0.033 | 0.038 | 0.003 | 0.004 |  |  |
| LR | 0.045 | 0.044 | 0.041 | 0.041 | 0.045 | 0.038 | 0.041 | 0.037 | 0.039 | 0.028 | 0.028 | 0.001 |  |
| HUR | 0.040 | 0.039 | 0.038 | 0.038 | 0.040 | 0.035 | 0.037 | 0.032 | 0.038 | 0.002 | 0.003 | 0.028 | 0.001 |

K2P distances were calculated from all 379 *cyt b* sequences representing the 13 populations. Off-diagonal values are mean between-population distances; diagonal values are mean within-population distances. Values are reported as substitutions per site and rounded to three decimal places; 0.000 may represent a small nonzero value.

Supplementary Table S3. Pairwise mitochondrial differentiation among *O. bidens* populations based on *cyt b*.

| Population pair | *F_st_* | 95% CI | Adjusted *P* |
| --- | --- | --- | --- |
| YZ1–YZ2 | 0.738 | [0.731, 0.891] | <0.001 |
| YZ1–YZ3 | 1.000 | [1.000, 1.000] | <0.001 |
| YZ1–YZ4 | 0.993 | [0.984, 1.000] | <0.001 |
| YZ1–YZ5 | 0.301 | [0.229, 0.426] | <0.001 |
| YZ1–YZ6 | 0.825 | [0.752, 0.887] | <0.001 |
| YZ1–YZ7 | 0.492 | [0.463, 0.580] | <0.001 |
| YZ1–PR | 0.988 | [0.981, 0.995] | <0.001 |
| YZ1–YR | 0.409 | [0.272, 0.569] | <0.001 |
| YZ1–SR | 0.990 | [0.986, 0.995] | <0.001 |
| YZ1–HAR | 0.952 | [0.904, 0.981] | <0.001 |
| YZ1–LR | 0.993 | [0.984, 1.000] | <0.001 |
| YZ1–HUR | 0.982 | [0.976, 0.990] | <0.001 |
| YZ2–YZ3 | 0.965 | [0.960, 0.988] | <0.001 |
| YZ2–YZ4 | 0.951 | [0.936, 0.978] | <0.001 |
| YZ2–YZ5 | 0.213 | [0.150, 0.420] | 0.002 |
| YZ2–YZ6 | 0.700 | [0.606, 0.796] | <0.001 |
| YZ2–YZ7 | 0.293 | [0.271, 0.395] | <0.001 |
| YZ2–PR | 0.942 | [0.932, 0.970] | <0.001 |
| YZ2–YR | 0.225 | [0.094, 0.400] | 0.035 |
| YZ2–SR | 0.967 | [0.960, 0.980] | <0.001 |
| YZ2–HAR | 0.906 | [0.838, 0.957] | <0.001 |
| YZ2–LR | 0.973 | [0.957, 0.989] | <0.001 |
| YZ2–HUR | 0.954 | [0.945, 0.970] | <0.001 |
| YZ3–YZ4 | 0.015 | NA | 0.112 |
| YZ3–YZ5 | 0.942 | [0.932, 0.957] | <0.001 |
| YZ3–YZ6 | 0.839 | [0.766, 0.897] | <0.001 |
| YZ3–YZ7 | 0.639 | [0.556, 0.758] | <0.001 |
| YZ3–PR | 0.989 | [0.983, 0.995] | <0.001 |
| YZ3–YR | 0.600 | [0.598, 0.671] | <0.001 |
| YZ3–SR | 0.989 | [0.985, 0.994] | <0.001 |
| YZ3–HAR | 0.949 | [0.898, 0.980] | <0.001 |
| YZ3–LR | 0.993 | [0.982, 1.000] | <0.001 |
| YZ3–HUR | 0.981 | [0.974, 0.989] | <0.001 |
| YZ4–YZ5 | 0.934 | [0.921, 0.952] | <0.001 |
| YZ4–YZ6 | 0.829 | [0.756, 0.889] | <0.001 |
| YZ4–YZ7 | 0.627 | [0.543, 0.742] | <0.001 |
| YZ4–PR | 0.981 | [0.970, 0.992] | <0.001 |
| YZ4–YR | 0.590 | [0.586, 0.663] | <0.001 |
| YZ4–SR | 0.985 | [0.979, 0.992] | <0.001 |
| YZ4–HAR | 0.944 | [0.893, 0.977] | <0.001 |
| YZ4–LR | 0.988 | [0.976, 0.998] | <0.001 |
| YZ4–HUR | 0.976 | [0.968, 0.986] | <0.001 |
| YZ5–YZ6 | 0.759 | [0.690, 0.826] | <0.001 |
| YZ5–YZ7 | 0.406 | [0.381, 0.494] | <0.001 |
| YZ5–PR | 0.917 | [0.905, 0.937] | <0.001 |
| YZ5–YR | 0.341 | [0.177, 0.526] | <0.001 |
| YZ5–SR | 0.959 | [0.953, 0.968] | <0.001 |
| YZ5–HAR | 0.919 | [0.872, 0.953] | <0.001 |
| YZ5–LR | 0.964 | [0.954, 0.975] | <0.001 |
| YZ5–HUR | 0.950 | [0.942, 0.962] | <0.001 |
| YZ6–YZ7 | 0.330 | [0.218, 0.503] | <0.001 |
| YZ6–PR | 0.397 | [0.314, 0.540] | <0.001 |
| YZ6–YR | 0.461 | [0.416, 0.550] | <0.001 |
| YZ6–SR | 0.892 | [0.843, 0.929] | <0.001 |
| YZ6–HAR | 0.847 | [0.775, 0.910] | <0.001 |
| YZ6–LR | 0.898 | [0.848, 0.937] | <0.001 |
| YZ6–HUR | 0.881 | [0.829, 0.920] | <0.001 |
| YZ7–PR | 0.485 | [0.379, 0.649] | <0.001 |
| YZ7–YR | 0.254 | [0.208, 0.395] | <0.001 |
| YZ7–SR | 0.792 | [0.745, 0.853] | <0.001 |
| YZ7–HAR | 0.750 | [0.683, 0.828] | <0.001 |
| YZ7–LR | 0.806 | [0.760, 0.866] | <0.001 |
| YZ7–HUR | 0.779 | [0.731, 0.843] | <0.001 |
| PR–YR | 0.561 | [0.560, 0.630] | <0.001 |
| PR–SR | 0.981 | [0.975, 0.988] | <0.001 |
| PR–HAR | 0.933 | [0.875, 0.971] | <0.001 |
| PR–LR | 0.986 | [0.973, 0.996] | <0.001 |
| PR–HUR | 0.971 | [0.962, 0.981] | <0.001 |
| YR–SR | 0.683 | [0.662, 0.775] | <0.001 |
| YR–HAR | 0.635 | [0.564, 0.739] | <0.001 |
| YR–LR | 0.680 | [0.643, 0.785] | <0.001 |
| YR–HUR | 0.669 | [0.647, 0.764] | <0.001 |
| SR–HAR | 0.116 | [0.083, 0.283] | <0.001 |
| SR–LR | 0.975 | [0.958, 0.989] | <0.001 |
| SR–HUR | 0.492 | [0.431, 0.597] | <0.001 |
| HAR–LR | 0.919 | [0.844, 0.969] | <0.001 |
| HAR–HUR | 0.128 | [0.087, 0.289] | <0.001 |
| LR–HUR | 0.964 | [0.945, 0.981] | <0.001 |

CI, bootstrap confidence interval; adjusted *P*, Benjamini–Hochberg-adjusted *P* value. NA indicates undefined *F_st_* in some bootstrap resamples.

Supplementary Table S4 Haplotype distribution in *O. bidens* populations based on *cyt b.*

| Groups | n | Haplotypes distribution |
| --- | --- | --- |
| YZ1 | 1 | Hap1(32) |
| YZ2 | 4 | Hap2(6),Hap3(1),Hap4(2),Hap5(1) |
| YZ3 | 1 | Hap6(31) |
| YZ4 | 3 | Hap6(27),Hap7(2),Hap8(1) |
| YZ5 | 6 | Hap1(7),Hap2(2),Hap9(7),Hap10(2),Hap11(4),Hap12(10) |
| YZ6 | 9 | Hap13(7),Hap14(1),Hap15(8),Hap16(1),Hap17(2),Hap18(1),Hap19(6),Hap20(2),Hap21(1) |
| YZ7 | 10 | Hap20(1),Hap22(12),Hap23(1),Hap24(2),Hap25(7),Hap26(1),Hap27(2),Hap28(1),Hap29(1),Hap30(1) |
| PR | 6 | Hap20(24),Hap31(1),Hap32(1),Hap33(3),Hap34(1),Hap35(1) |
| YR | 3 | Hap11(4),Hap36(14),Hap37(11) |
| SR | 10 | Hap38(20),Hap39(1),Hap40(1),Hap41(2),Hap42(3),Hap43(1),Hap44(1),Hap45(1),Hap46(1),Hap47(1) |
| HAR | 11 | Hap38(4),Hap48(4),Hap49(4),Hap50(8),Hap51(1),Hap52(1),Hap53(3),Hap54(1),Hap55(1),Hap56(4),Hap57(1) |
| LR | 3 | Hap58(27),Hap59(2),Hap60(1) |
| HUR | 7 | Hap50(16),Hap61(9),Hap62(2),Hap63(1),Hap64(2),Hap65(1),Hap66(1) |

Supplementary Table S5 Mean pairwise nucleotide differences and Ka/Ks values of the 13 PCGs in *O. bidens.*

| PCGs | Mean pairwise nucleotide difference % | Ka/Ks |
| --- | --- | --- |
| *atp6* | 2.572 | 0.006 |
| *atp8* | 1.361 | 0.056 |
| *cox1* | 1.637 | 0.000 |
| *cox2* | 1.757 | 0.019 |
| *cox3* | 1.352 | 0.023 |
| *nd1* | 4.295 | 0.027 |
| *nd2* | 5.204 | 0.057 |
| *nd3* | 2.564 | 0.001 |
| *nd4* | 3.497 | 0.046 |
| *nd4L* | 3.768 | 0.045 |
| *nd5* | 3.136 | 0.037 |
| *nd6* | 4.533 | 0.049 |
| *cyt b* | 2.743 | 0.002 |
| Concatenated 13 PCGs | 3.007 | — |
| Concatenated 12 PCGs excluding *cyt b* | 3.034 | — |

Nucleotide differences are expressed as mean uncorrected p-distances (%). Ka/Ks denotes the ratio of nonsynonymous to synonymous substitution rates; “—” indicates not estimated. Values shown as 0.000 are rounded.

Supplementary Table S6 The base composition and base preference of mitochondrial genomes in different geographical populations of *O. bidens.*

| ID | Full sequence | | | | | PCGs | | | | |
| --- | --- | --- | --- | --- | --- | --- | --- | --- | --- | --- |
|  | length (bp) | AT(%) | GC(%) | AT skewness | GC skewness | length (bp) | AT(%) | GC(%) | AT skewness | GC skewness |
| YZ1 | 16,611 | 53.8 | 46.2 | 0.004 | -0.173 | 11,439 | 52.8 | 47.2 | -0.096 | -0.181 |
| YZ2 | 16,611 | 53.8 | 46.2 | 0.004 | -0.174 | 11,439 | 52.8 | 47.2 | -0.095 | -0.182 |
| YZ3 | 16,612 | 53.7 | 46.3 | 0.006 | -0.175 | 11,439 | 52.7 | 47.2 | -0.093 | -0.182 |
| YZ4 | 16,612 | 53.7 | 46.3 | 0.005 | -0.174 | 11,439 | 52.8 | 47.2 | -0.094 | -0.181 |
| YZ5 | 16,611 | 53.8 | 46.2 | 0.004 | -0.173 | 11,439 | 52.8 | 47.2 | -0.095 | -0.181 |
| YZ6 | 16,611 | 53.9 | 46.2 | 0.009 | -0.180 | 11,403 | 52.8 | 47.2 | -0.086 | -0.192 |
| YZ7 | 16,611 | 53.8 | 46.2 | 0.004 | -0.174 | 11,346 | 52.9 | 47.1 | -0.097 | -0.180 |
| PR | 16,611 | 53.8 | 46.2 | 0.008 | -0.179 | 11,343 | 52.9 | 47.1 | -0.082 | -0.193 |
| YR | 16,611 | 53.8 | 46.2 | 0.005 | -0.174 | 11,223 | 52.9 | 47.2 | -0.086 | -0.191 |
| SR | 16,610 | 53.7 | 46.3 | 0.006 | -0.178 | 11,457 | 52.8 | 47.1 | -0.088 | -0.188 |
| HAR | 16,610 | 53.7 | 46.2 | 0.006 | -0.178 | 11,421 | 52.9 | 47.1 | -0.089 | -0.188 |
| LR | 16,613 | 53.9 | 46.1 | 0.005 | -0.177 | 11,334 | 53.1 | 46.9 | -0.085 | -0.194 |
| HUR | 16,610 | 53.8 | 46.3 | 0.008 | -0.180 | 11,361 | 52.8 | 47.2 | -0.083 | -0.191 |
